# Differential transcriptome analysis of glandular and filamentous trichomes in *Artemisia annua*

**DOI:** 10.1186/1471-2229-13-220

**Published:** 2013-12-20

**Authors:** Sandra SA Soetaert, Christophe MF Van Neste, Mado L Vandewoestyne, Steven R Head, Alain Goossens, Filip CW Van Nieuwerburgh, Dieter LD Deforce

**Affiliations:** 1 Laboratory of Pharmaceutical Biotechnology, Faculty of Pharmaceutical Sciences, Ghent University, Harelbekestraat 72, 9000 Ghent, Belgium; 2 Department of Plant Systems Biology, VIB and Department of Plant Biotechnology and Bioinformatics, Ghent University, Technologiepark 927, 9052 Ghent, Belgium; 3 Next Generation Sequencing Core, The Scripps Research Institute, 10550N. Torrey Pines Rd, La Jolla, CA 92037 United States of America

**Keywords:** *Artemisia annua*, Artemisinin, RNASeq, Glandular trichomes, Filamentous trichomes, Laser microdissection pressure catapulting, MEP pathway, Mevalonate pathway, Lipid biosynthesis, Terpene biosynthesis

## Abstract

**Background:**

The medicinal plant *Artemisia annua* is covered with filamentous trichomes and glandular, artemisinin producing trichomes. A high artemisinin supply is needed at a reduced cost for treating malaria. Artemisinin production in bioreactors can be facilitated if a better insight is obtained in the biosynthesis of artemisinin and other metabolites. Therefore, metabolic activities of glandular and filamentous trichomes were investigated at the transcriptome level.

**Results:**

By laser pressure catapulting, glandular and filamentous trichomes as well as apical and sub-apical cells from glandular trichomes were collected and their transcriptome was sequenced using Illumina RNA-Seq. A *de novo* transcriptome was assembled (Trinity) and studied with a differential expression analysis (edgeR).

A comparison of the transcriptome from glandular and filamentous trichomes shows that MEP, MVA, most terpene and lipid biosynthesis pathways are significantly upregulated in glandular trichomes. Conversely, some transcripts coding for specific sesquiterpenoid and triterpenoid enzymes such as 8-epi-cedrol synthase and an uncharacterized oxidosqualene cyclase were significantly upregulated in filamentous trichomes. All known artemisinin biosynthesis genes are upregulated in glandular trichomes and were detected in both the apical and sub-apical cells of the glandular trichomes. No significant differential expression could be observed between the apical and sub-apical cells.

**Conclusions:**

Our results underscore the vast metabolic capacities of *A. annua* glandular trichomes but nonetheless point to the existence of specific terpene metabolic pathways in the filamentous trichomes. Candidate genes that might be involved in artemisinin biosynthesis are proposed based on their putative function and their differential expression level.

## Background

*Artemisia annua* L. (Sweet Wormwood) is a medicinal plant that produces artemisinin which is a sesquiterpene with anti-malarial properties. Since every year around 216 million people are infected with malaria [1], a high supply of artemisinin is needed at a reduced cost. Artemisinin production is being enhanced in *A. annua* by crossing high-producing plants [2]. Another strategy to increase production of artemisinin is synthesis of artemisinic-acid in engineered yeast and subsequent (photo)chemical conversion to artemisinin [3,4]. A better insight in artemisinin biosynthesis could lead to a cheaper production method.

**Figure 1:**
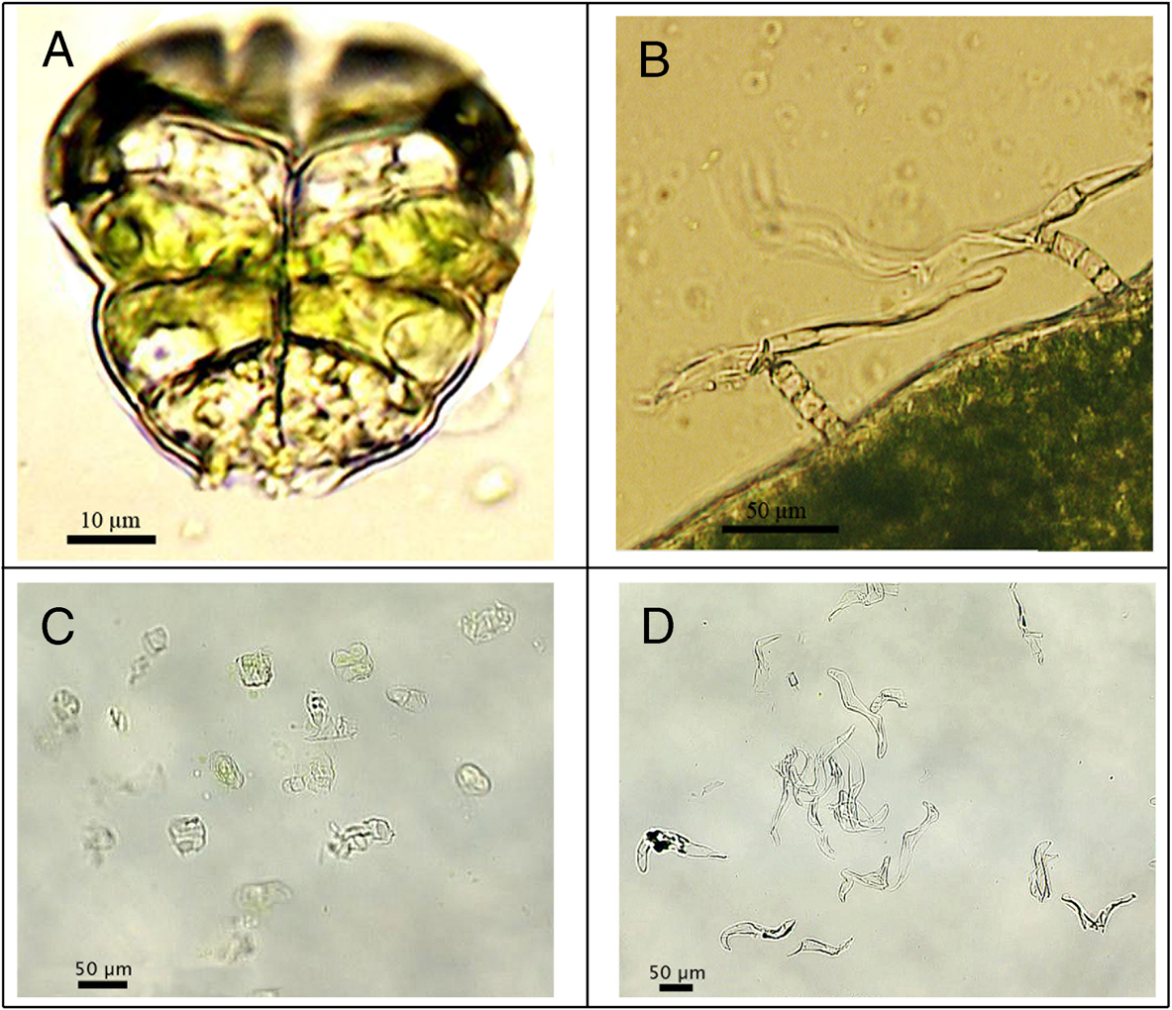
**Glandular and filamentous trichomes of *A. annua*. A**: Glandular trichome with on top a pair of white apical cells and two pairs of green sub-apical cells surrounded by a sub-cuticular cavity (cells at the basis of the trichome are partially removed); **B**: Filamentous trichomes (T-shape) with stalk cells and elongated cell; **C**: Glandular trichomes captured by laser pressure catapulting in the cap of a tube; **D**: Filamentous trichomes captured by laser pressure catapulting in the cap of a tube.

An important breakthrough unravelling artemisinin biosynthesis, was the localization of artemisinin production in the glandular trichomes [5]. Trichomes, named after the Greek word for hair are epidermal outgrowths covering plant organs. In *A. annua*, two types of trichomes are present: biseriate peltate glandular trichomes (Figure 1A) and filamentous trichomes composed of stalk cells and an elongated cell in a T-shape (Figure 1B) [6]. Duke *et al*. compared a normal biotype of *A. annua* with both filamentous and glandular trichomes to a biotype with only filamentous trichomes. Only in the presence of glandular trichomes, artemisinin was detected [5].

This information was used to discover candidate genes for artemisinin biosynthesis with an expressed sequence tag (EST) approach [7]. Three EST libraries were constructed: glandular trichome, flower bud and glandular trichome-minus-flower-bud subtracted library. Several genes that were preferentially expressed in glandular trichomes are involved in artemisinin biosynthesis such as *CYP71AV1, ALDH1, DBR2* and *ADH1*[7-10].

Enzymes involved in artemisinin biosynthesis are known up to the formation of dihydroartemisinic acid. It is not yet clear whether the last step(s) from dihydroartemisinic acid to artemisinin involves a spontaneous auto-oxidation or is catalyzed by enzymes. Brown and Sy favour the theory of spontaneous chemistry because they see parallels between *in vitro* auto-oxidation and intermediates present *in vivo*[11]*.* Additionally, plants fed with labelled dihydroartemisinic acid and dried, contained the same proportion of labelled artemisinin as plants that were kept alive [11]. These are the main arguments for chemical conversion. On the other hand, while 70% of label incorporation was detected in the metabolites derived from dihydroartemisinic acid; artemisinin and arteannuin H had only 5-15% label incorporation [11]. Therefore, it cannot be excluded that there is another more important pathway leading to artemisinin which was not accessible by the labelled precursor and that enzymes might be involved to catalyze this process in *A. annua*.

To find candidate genes that catalyze the last step(s) to artemisinin, a more detailed analysis was needed of the transcriptome of glandular trichomes. Sequencing of the transcriptome from enriched glandular trichome preparations from *A. annua* was performed by Graham *et al*. on the Roche 454 platform to identify genes and markers for fast-track breeding [2]. In another study, Wang *et al*. performed a global transcriptome characterization of glandular trichomes. To confirm the expression of some genes in glandular trichomes a semi-quantitative RT-PCR analysis was performed on filamentous and glandular trichomes. Three genes involved in terpene biosynthesis were tested: amorpha-4,11-diene synthase, a sesquiterpene cyclase and (3R)-linalool synthase. They found that these genes were expressed in glandular trichomes as well as in filamentous trichomes [12] and this raised the question whether filamentous trichomes are involved in the production of secondary metabolites.

Trichomes are in general classified as non-glandular (e.g. filamentous trichomes in *A. annua*) or glandular, based on their secretory capacity. In plants, glandular trichomes are most often production sites for multiple secondary metabolites which form a first-line defence at the surface of the plant through their capacity to entrap, deter or poison pathogens and herbivores [13]. Because of their interesting metabolite content as illustrated with artemisinin, a large number of studies have been devoted to glandular trichomes.

In contrast to the extensive literature describing glandular trichomes, less attention has been paid to non-glandular trichomes. Non-glandular trichomes are assumed to form a physical barrier by steric hindrance of herbivores [14,15]. Non-glandular trichomes are mainly described for taxonomic and phylogenetic purposes [16-20] but little is known about their production of secondary metabolites [21].

To detect potential candidate genes for artemisinin biosynthesis and to investigate if filamentous trichomes produce important secondary metabolites, it is interesting to compare filamentous and glandular trichomes. Therefore, we performed a comparative transcriptome analysis of filamentous and glandular trichomes on the Illumina HiSeq platform. Several cytochromes, peroxidases and dioxygenases that are potentially involved in the biosynthesis of artemisinin and/or other terpenes were upregulated in glandular trichomes. Our transcriptome analysis confirms the established metabolic capacities of *A. annua* glandular trichomes but also points to specific metabolic activities in *A. annua* filamentous trichomes.

Additionally, two other transcriptome experiments were set up to discover potential candidate genes. First, the effect of jasmonic acid (JA) elicitation on glandular and filamentous trichomes was investigated as e.g. Maes *et al*. showed that artemisinin production can be stimulated by JA [22].

In another experiment, the transcriptome of two cell types from glandular trichomes was investigated to examine in which cells the production of artemisinin, and/or other secondary metabolites occurs. Glandular trichomes contain morphologically distinct types of secretory cells: white apical cells and green-coloured sub-apical cells. In both cell types, Olofsson *et al*. detected expression of genes involved in artemisinin biosynthesis [23]. In our data set, artemisinin biosynthesis genes were detected in both cell-types and no significant conclusion could be drawn about differential expression.

## Methods

### Overview of collected samples

In total 6 sample-types have been collected from capitula (flower heads) of *A. annua* (Table 1)*.* Three independent repeats of glandular (Figure 1C) and filamentous (Figure 1D) trichomes were collected from the same mock- and JA-treated plants. In addition to this, two repeats of separated apical and sub-apical cells of glandular trichomes were collected from mock-treated plants. The RNA from one of the 2 repeats for each cell type was split in two and separately amplified, creating 3 repeats for sequencing.

**Table 1:** Overview of collected samples

**Sample-type**	**Apical cells**		**Sub-apical cells**		**Glandular trichomes**		**Filamentous trichomes**	
**Treatment**	**Mock**		**Mock**		**Mock**	**JA**	**Mock**	**JA**
RNA extract 1	Unfixated		Unfixated		Unfixated Rep1	Unfixated Rep1	Unfixated Rep1	Unfixated Rep1
Amplification	ampl 1	ampl 2	ampl 1	ampl 2	ampl	ampl	ampl	ampl
RNA extract 2	Fixated		Fixated		Unfixated Rep2	Unfixated Rep2	Unfixated Rep2	Unfixated Rep2
Amplification	ampl		ampl		ampl	ampl	ampl	ampl
RNA extract 3					Unfixated Rep3	Unfixated Rep3	Unfixated Rep3	Unfixated Rep3
Amplification					ampl	ampl	ampl	ampl

Overview of collected samples from *A. annua*. Unfixated glandular and filamentous trichomes are collected from mock- and JA-treated samples in 3 repeats (Rep). For apical and sub-apical cells, one sample of unfixated and one sample of fixated cells are collected. The unfixated sample was amplified in 2 separate amplifications (ampl).

### Plant preparation

Experiments were executed on *Artemisia annua* L. Anamed A3 cultivar (http://www.anamed.net). This cultivar contains up to 1,4% artemisinin (dry weight leaves) and is the result of cross breeding high-artemisinin producing plants by Mediplant Inc. (Conthey, Switzerland) [24]. Anamed A3 can grow well in tropical regions and is not as photosensitive as other breeds [25]. Under 8 hours day and 16 hours night, Anamed A3 starts flowering at the earliest after 6 months [see Additional file 1]. The length of this pre-flowering period is in line with observations under field conditions [26].

Seeds from *A. annua* Anamed were sterilized for 2 min in 70% EtOH (Merck, Darmstadt, Germany) and 10 min in a solution with 3.84 ml NaOCl (10-13% chlorine from Sigma-Aldrich, Steinheim, Germany), 5 μl Tween 20 (MP Biomedicals, Illkirch, France) and 6.16 ml sterile water. Subsequently, seeds were rinsed with sterile water and germinated on moist paper. After 1 or 2 weeks, shoots were transferred to soil and grown under a regime with 8 hours day, 16 hours night and a temperature of 20°C. After 6 months, flowers were appearing. For JA elicitation, plants were treated for 8 days before the start of the sampling procedure by spraying every 2 days with a solution of 100 μM JA (Duchefa, Haarlem, The Netherlands) containing 1.5 mM Tween20 (MP Biomedicals) and adding every 2 days 5 ml of 100 μM JA to the soil. During the sampling procedure, treatment was continued every 2 days. Control groups were treated with a mock (water) solution in a separate room.

### Glandular and filamentous sample preparation

On the basal bracts and pedicel of the capitulum, filamentous trichomes are abundantly present [6]. Glandular trichomes are protruding on the corolla of the floret buds from the capitulum but are sunken in the capitulum bracts and in leaves [6]. An image of a flower head from *A. annua* Anamed was taken with Tabletop SEM (TM-1000, Hitachi, Tokyo, Japan) [see Additional file 2]. Sunken trichomes are difficult to collect with laser capture microdissection and therefore, capitula were used to collect glandular and filamentous trichomes. Trichomes were collected from mock and JA-treated plants in 3 independent biological repeats. For each repeat, trichomes were collected from a pool of 3 plants. The same plants were used for capturing 190 glandular and 670 filamentous trichomes.

On a RNA free microscope slide, a capitulum was cut in a drop of cold buffer with 25 mM MOPSO (pH6.3) (Sigma-Aldrich), 200 mM sorbitol (Alfa Aesar, Karlsruhe, Germany), 10 mM sucrose (Acros, Geel, Belgium), 5 mM thiourea (Sigma-Aldrich), 2 mM DTT (Sigma-Aldrich), 5 mM MgCl_2_ (Sigma-Aldrich) and 0.5 mM sodium-phosphate (Acros) [23]. Trichomes were captured, using the Palm MicroBeam system (P.A.L.M Microlaser Technologies, München, Germany) with a nitrogen UV-A laser (wavelength 355 nm). The laser-beam was focussed on the tissue for laser microdissection, to separate trichomes or cells from the surrounding tissue. Free trichomes were thereafter captured with laser pressure catapulting by focussing the laser beam just below the tissue. Samples were collected in 30 μl lysis buffer with ß-mercaptoethanol (Absolutely RNA Nanoprep Kit Stratagene, La Jolla, CA). An image of collected glandular and filamentous trichomes is shown in Figure 1C and D. RNA was extracted with the Absolutely RNA Nanoprep protocol with DNase treatment. Samples were eluted in 10 μl and half was used as starting material for RNA amplification.

### Sample preparation of apical and sub-apical cells

Apical and sub-apical cells were collected from mock-treated plants in the same way as described for glandular and filamentous trichomes. In a first set of samples, 300 unfixated cells of each cell type were collected. In a second set, 500 fixated cells of each cell-type were collected. In this second set, the cells were fixated before laser microdissection and catapulting to make the separation of the cells easier. Fixation of flowers was carried out by subjecting the samples to a 4% formaldehyde phosphate buffered saline solution for 3 to 4 hours in vacuum. RNA was extracted with the Absolutely RNA Nanoprep protocol with DNase treatment. The RNA from the unfixated samples was split in two and each subsample was separately amplified to create a third sample set for sequencing.

### RNA amplification and sequencing

Samples were amplified with a linear amplification system: the Ovation RNA-Seq System with 1 h 30 min Spia-amplification (NuGen, AC Bemmel, The Netherlands). In this amplification procedure, random primers and oligo dT primers are used during RNA amplification and consequently the 5’ end of the mRNA is better amplified compared to when only oligo dT primers are used. The cDNA priming reaction used in the NuGEN amplification kits are designed to avoid amplification of rRNA sequences. After RNA amplification, barcoded Illumina sequencing libraries were made using a post amplification ligation-mediated strategy [27]. The 18 samples were sequenced with a read-length of 100 bp in 3 lanes of an Illumina HiSeq 2000 flowcell.

### Quantitative real time (qRT)-PCR

Nugen amplified DNA from 3 independent mock-treated glandular and filamentous trichome samples was analyzed with qRT-PCR. As template, 2 ng DNA was used in 10 μl reactions containing 5 μl iTaq SYBR Green Supermix with ROX (Bio-Rad, Watford, UK) and 400 nM primers. The qRT-PCR experiment was performed on a Light Cycler 480 (Roche) with hotstart at 95°C for 2 min. and 42 cycles 95°C (15 sec.), 52°C (1 min.), including melting curve analysis. Each qRT-PCR reaction was executed in duplo and these technical repeats were averaged prior to qbasePLUS (version 2.3) analysis with normalization to input DNA concentration [28]. To validate this normalization strategy, 3 genes were included that are expected to be similarly expressed in both trichome types: Actin 2 (homologue *AT3G18780*), protein phosphatase 2A subunit A3 (*PP2AA3*; homologue *AT1G13320*) and a pentatricopeptide repeat (PPR) superfamily protein (homologue *AT5G55840*). These genes were selected based on *Arabidopsis* data from Czechowski *et al*. [29] and expression of homologous transcripts in our RNASeq experiment. Other transcripts analyzed were artemisinin-synthesis and triterpene-synthesis related. Primers [see Additional file 3] were adopted from other manuscripts [22,30] or designed with Primer-BLAST from NCBI [31].

### Trinity and Blast2GO

A *de novo* transcriptome was assembled from all 18 samples. Trinity Release-2011-07-13 was used to perform an ALLPATHS error correction [32] on the reads prior to the *de novo* assembly which was made with Trinity Release-2011-08-20 [33]. The 150,288 Trinity contigs were annotated using BLAST and the NCBI non-redundant protein database. The best 5 BLAST hits were used to indicate a putative function. The Blast2GO suite [34] was used to generate gene ontology terms based on the BLAST output. Two sets of settings were sequentially used: strict and more permissive. In the more strict settings, a BLAST high-scoring segment pairs (hsp) length of 33 and a minimum hsp coverage of the query of 33 was required. The more permissive setting allowed for a shorter BLAST hsp length of 20 with no minimum hsp coverage of the query.

### Bowtie and RSEM

Bowtie [35] and RSEM (RNASeq by Expectation Maximization) [36] were used for mapping the 289 million reads to the Trinity *de novo* assembly and counting the number of reads that matched to each contig. Standard options were used, but RSEM’s polyA tail option was disabled.

### edgeR

To perform the differential expression analysis, an R script was developed that makes use of the Bioconductor edgeR package [37]. All glandular and filamentous samples were normalized together. Normalization was performed by trimmed mean of M values (TMM). TMM equates the overall expression levels of genes between samples under the assumption that the majority of them are not differentially expressed [38]. A p-value < 0.05, adjusted for multiple testing, was used to determine which contigs could be differentially expressed. The edgeR parameter prior.n was set to 1.

### MapMan

MapMan is a program to visualize various pathways and to indicate genes or contigs that are up or down-regulated [39]. Prior to MapMan, functional bins need to be assigned to contigs of the *de novo* assembly. To assign the bins, Mercator was used (default options, Blast_cutoff: 50 and IS_DNA). Not all artemisinin biosynthesis genes were automatically assigned to a bin. For those genes, additional bins were added based on the definitions of the best 5 blast hits. The output of edgeR was visualized with MapMan to show which metabolic pathways are significantly differentially expressed.

## Results and discussion

### *De novo* assembly of the transcriptome

All 18 samples were sequenced in 3 lanes of an Illumina HiSeq 2000 flowcell. For each sample, on average 16 million single-end reads of 100 bp were generated. Around 60% of the reads had hits with a combined LSU and SSU rRNA reference database [40] and tRNA database with all known tRNAs [41]. The total of 289 million reads generated were processed using Trinity [33] to assemble a *de novo* transcriptome [see Additional file 4] containing 150,288 contigs in 108,400 homologous groups with an average contig-length of 412 bp and a minimum and maximum length of 201 and 7775 bp, respectively (Table 2).

**Table 2:** Length distribution of Trinity contigs

**Contig length (bp)**	**Number of contigs**
< 500	118556
500-1000	24993
1000-1500	4658
1500-2000	1299
2000-3000	643
> 3000	133

To have a better estimate of the quality of the Trinity contigs, all 88 full-length *A. annua* mRNA sequences available in the NCBI non-redundant protein database were compared with the Trinity contigs. Of those, only 2 had no BLAST hits to the Trinity contigs. Each trinity contig that showed a hit with one of the NCBI sequences, covered on average 58% of the length of the NCBI sequence. A combination of contigs with the same BLAST-hit could together cover on average 84% of the NCBI sequence. If the set of NCBI sequences is assumed to be representational of the real transcripts, one can expect that more than 80% of the length of a random transcript is present in our assembly.

### Annotation and characterization of *de novo* transcripts

Eighty-four% of the 150,288 contigs showed at least one BLAST hit with the NCBI non-redundant protein database. The first five BLAST hits served as an indication for the putative function of a contig. The contigs were also annotated with the Blast2GO suite [34], which links BLAST hits to gene ontology (GO) terms: 46,711 contigs had a good connection to GO terms, using strict settings for Blast2GO; 5,596 contigs still had a connection to GO terms with more permissive settings of Blast2GO; the remaining 73,389 contigs with BLAST hits did not point to any GO terms. As *A. annua* is not a model organism and does not belong to a family of a model organism it is logical that a lot of contigs with BLAST hits were not annotated with GO terms. It is mostly for genes that show strong homologies with genes of model organism that one can expect a successful annotation using this strategy. Moreover, a gene that performs a different function but has a homology with a gene of a model organism, can be annotated wrongly.

To further characterize our *de novo* Illumina transcripts, they were also compared to the 454 glandular trichome *A. annua* contigs of Wang *et al*. [12]. Of their 42,678 contigs, 79% had a BLAST hit within our Trinity contigs. Vice versa, only 20% of our contigs had a BLAST hit within their contig set. This was not due to contamination of rRNA and tRNA reads that could have resulted from our method of mRNA amplification since only 0.6% of all our contigs showed a hit with rRNA or tRNA. Therefore the fact that the majority of our contigs are differential from those of Wang *et al*. is most likely the result from a greater coverage.

### Influence of JA treatment

The transcriptome of mock and JA-treated trichome samples was compared and no significant differences (adjusted p-value < 0.05) were detected. Accordingly, at the metabolite level, equal amounts of artemisinin, arteannuin B, dihydroartemisinic acid and artemisinic acid were measured with HPLC-MS/MS as shown in additional data [see Additional file 5 and Additional file 6]. This indicates that the JA-treatment did not have a major influence in our experimental set-up. This was not expected since Maes *et al*. measured higher artemisinin levels after JA treatment [22]. A possible explanation is the use of plants in different developmental stages: Maes *et al*. used young seedlings while in our RNASeq experiment 6-months old plants with closed capitula were used. It has been shown that in flowers of *Arabidopsis thaliana*, JA levels increase 6.7-fold just before flower bud opening [42,43]. Therefore it is plausible that endogenous JA-signalling already reached a maximum effect in our samples and that exogenous JA treatment did not trigger an additional response.

### Glandular versus filamentous trichomes

Transcript levels from glandular trichomes and filamentous trichomes were compared to obtain a list of significantly differentially expressed contigs (adjusted p-value < 0.05). To make a robust statistical comparison, 3 samples of glandular trichomes with JA and 3 samples with mock-treatment were compared to the 6 samples (mock and JA) of filamentous trichomes. Of 150,288 contigs; 631 were significantly differentially expressed and all these contigs are listed in additional data [see Additional file 7]. From these, 204 contigs were more expressed in filamentous trichomes whereas, 427 contigs were more expressed in glandular trichomes. An overview with contigs discussed in this article and their normalized counts for each sample, log_2_-fold changes and adjusted p-values are shown in additional data [see Additional file 8].

### MVA and MEP pathway in glandular and filamentous trichomes

From the upregulated contigs, 5% in glandular and 0% in filamentous trichomes were involved in the mevalonate (MVA) or 2-C-methyl-D-erythritol 4-phosphate (MEP) pathways. The MEP and MVA pathways produce isopentenyl diphosphate and its isomer dimethylallyl diphosphate, which are precursors for the production of terpenes.

**Figure 2:**
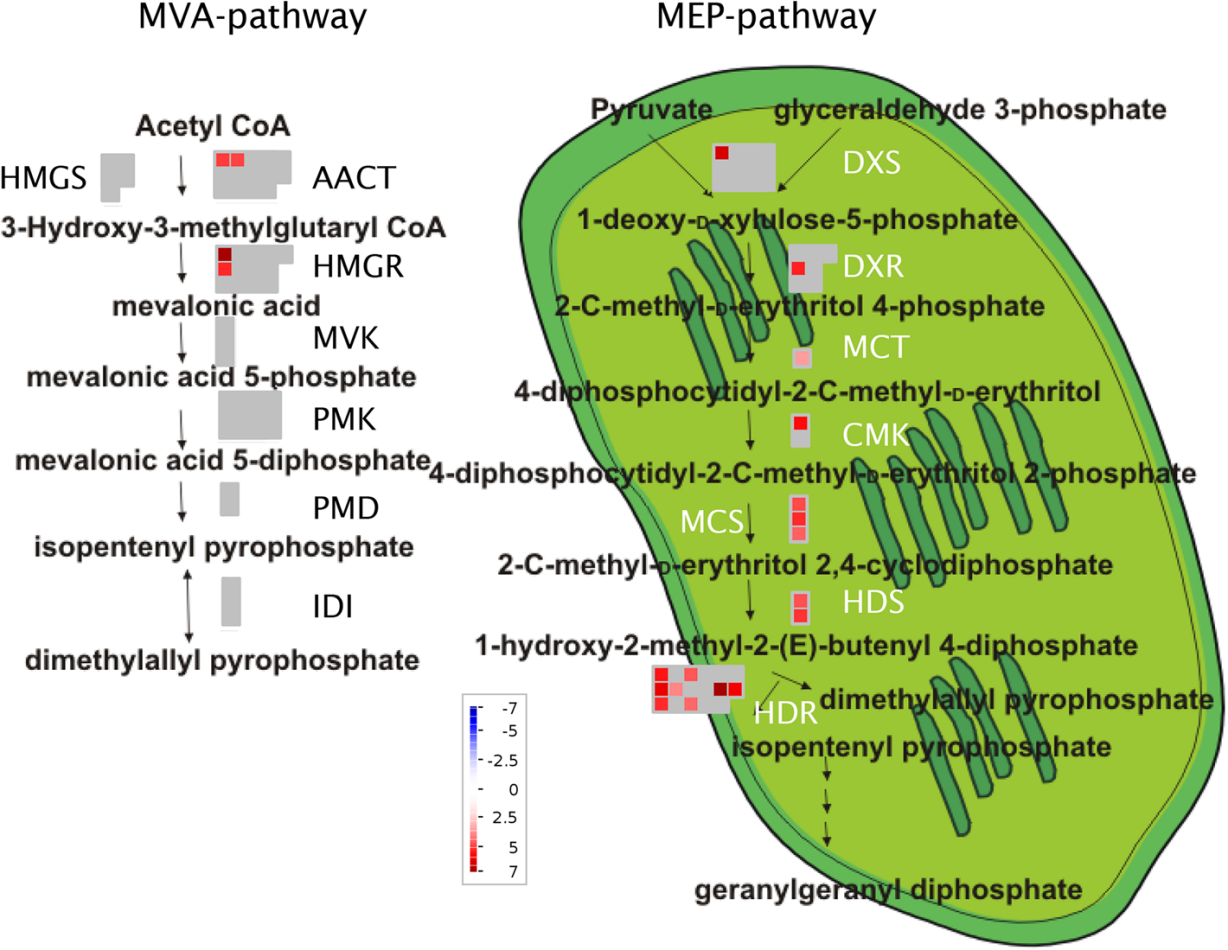
**Differentially expressed MVA and MEP-pathway genes in glandular and filamentous trichomes.** MapMan figure (adapted from [46]) with comparison of MVA and MEP pathways in glandular and filamentous trichomes. Significantly more expressed contigs in glandular trichomes are shown in red with colour scale to indicate the log_2_-fold changes. No contigs were significantly more expressed in filamentous trichomes. Grey represents contigs that were not significantly differentially expressed. MVA pathway: acetyl-CoA C-acetyltransferase (AACT), 3-hydroxy-3-methyl-glutaryl coenzyme A synthase (HMGS), 3-hydroxy-3-methyl-glutaryl coenzyme A reductase (HMGR), mevalonate kinase (MVK), phosphomevalonate kinase (PMK), diphosphomevalonate decarboxylase (PMD), isopentenyl diphosphate isomerase (IDI). MEP pathway: 1-deoxy-D-xylulose-5-phosphate synthase (DXS), 1-deoxy-D-xylulose-5-phosphate reductoisomerase (DXR), 2-C-methyl-D-erythritol-4-phosphate cytidylyltransferase (MCT), 4-cytidine 5’-diphospho-2-C-methyl-D-erythritol kinase (CMK), 2-C-methyl-D-erythritol-2,4-cyclodiphosphate synthase (MCS), hydroxy-2-methyl-2-(E)-butenyl 4-diphosphate synthase (HDS) and hydroxy-2-methyl-2-(E)-butenyl 4-diphosphate reductase (HDR).

MEP and MVA pathways were detected in glandular and filamentous trichomes. All transcripts coding for enzymes of the MEP pathway were significantly upregulated in glandular trichomes (Figure 2). From the MVA pathway, only *acetyl-CoA C-acetyltransferase (AACT)* and *3-hydroxy-3-methylglutaryl coenzyme A reductase (HMGR)* were significantly upregulated in glandular trichomes (Figure 2). Up-regulation of *HMGR* in glandular trichomes is important since it is shown that HMGR activity limits artemisinin biosynthesis [44,45].

### Artemisinin biosynthesis in glandular and filamentous trichomes

**Figure 3:**
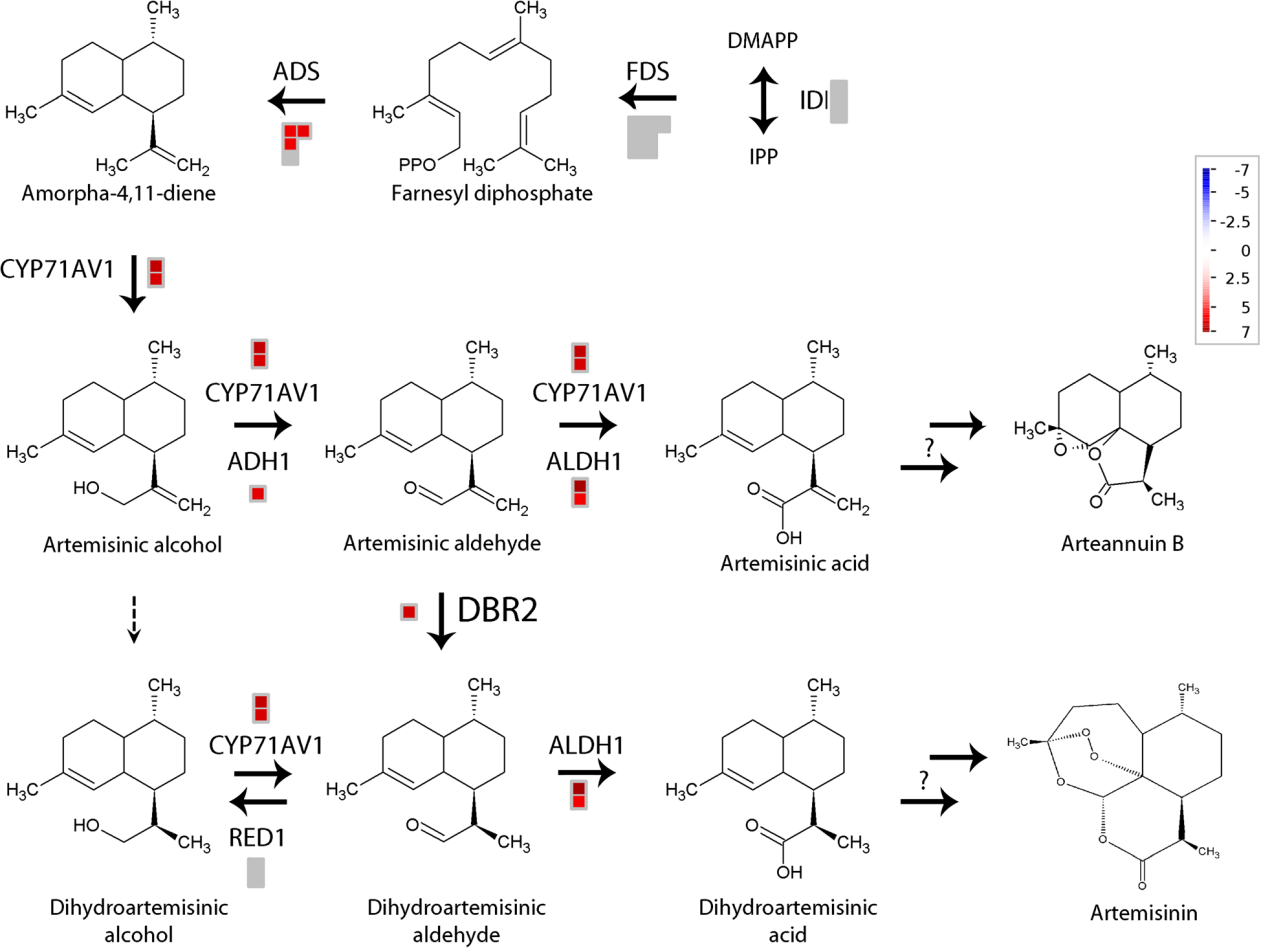
**Differentially expressed artemisinin biosynthesis genes in glandular and filamentous trichomes.** MapMan file with comparison of artemisinin biosynthesis pathways in glandular and filamentous trichomes. Significantly more expressed contigs in glandular trichomes are shown in red with colour scale to indicate the log_2_-fold change. There were no contigs with higher expression in filamentous trichomes. Grey represents contigs that were not significantly differentially expressed.

Terpene synthesis genes were accounting for 6% of the upregulated contigs in glandular trichomes and only 1% in filamentous trichomes. Starting from the MVA and MEP pathway, farnesyl diphosphate is synthesised by farnesyl diphosphate synthase (FDS) whose transcripts were not significantly upregulated in glandular trichomes (Figure 3 and Additional file 8). Subsequently, farnesyl diphosphate is converted to amorpha-4,11-diene which is the starting product for artemisinin biosynthesis. This reaction is catalyzed by amorpha-4,11-diene synthase (ADS) [47] and transcripts coding for this enzyme were detected more in glandular trichomes . By CYP71AV1, amorpha-4,11-diene is converted to artemisinic alcohol [3,7]. Thereafter, artemisinic alcohol is oxidized by CYP71AV1 [3,7] and alcohol dehydrogenase 1 (ADH1) [10] to artemisinic aldehyde. Artemisinic aldehyde is further oxidized by aldehyde dehydrogenase 1 (ALDH1) [8] and CYP71AV1 [3,7] to artemisinic acid or reduced by artemisinic aldehyde Δ11(13) double bond reductase (DBR2) [9] to form dihydroartemisinic aldehyde. A broad substrate oxidoreductase (RED1) can convert dihydroartemisinic aldehyde to dihydroartemisinic alcohol [48]. This reaction competes with ALDH1 using dihydroartemisinic aldehyde to form dihydroartemisinic acid [8]. Dihydroartemisinic acid is considered to be the precursor leading to artemisinin [9,49,50]. Transcripts corresponding to all these enzymes involved in the conversion of amorpha-4,11-diene to dihydroartemisinic acid were significantly upregulated in glandular trichomes, except for *RED1* (Figure 3).

These results confirm previous data pointing to glandular trichomes as the major artemisinin production site. A short dip in chloroform causes the collapse of the sub-cuticular space of glandular trichomes and extracts almost all artemisinin [5,51]. In addition to this, no artemisinin has been detected in a biotype of *A. annua* with only filamentous trichomes [5]. Nevertheless, in all samples of filamentous trichomes, transcripts corresponding to known artemisinin biosynthesis genes were detected, albeit at very low levels [see Additional file 8]. This is in agreement with previous reports, in which expression of *ADS* was also detected by RT-PCR in filamentous trichomes [12]. It should be noted though that in contrast, no staining of filamentous trichomes was observed in a promoter-GUS fusion study with the *ADS* promoter [52].

### Candidate genes for artemisinin biosynthesis

Contigs that are upregulated in glandular trichomes are possibly linked to artemisinin biosynthesis genes. The subset of these contigs which were annotated as cytochromes, peroxidases and dioxygenases might be possible candidates for the endoperoxide ring formation in artemisinin. An overview of these contigs is given in Table 3, more results per repeat are given in additional data [see Additional file 8].

**Table 3:** Potential candidate genes for artemisinin biosynthesis

**Annotation**	**Contig**	**Sum normalized counts of filamentous trichomes**	**Sum normalized counts of glandular trichomes**	**Log**_**2**_**fold change**	**Adjusted p-value**
**Cytochromes**					
CYP81	comp69_c0_seq1_1	1302	77238	5.9E + 00	8.2E-05
CYP82	comp548_c0_seq1_1	106	4142	5.2E + 00	3.5E-03
	comp548_c0_seq2_1	128	3547	4.8E + 00	5.0E-02
	sum	234	7689		
CYP76B1	comp15043_c0_seq2_1	0	233	3.2E + 01	4.6E-02
CYP450	comp3673_c0_seq1_1	18	1368	6.4E + 00	2.4E-02
cytochrome c-type	comp586_c0_seq1_1	28	3002	6.4E + 00	1.3E-03
	comp586_c0_seq3_1	26	4256	7.4E + 00	3.5E-05
	comp586_c0_seq4_1	0	106	3.1E + 01	2.5E-02
	sum	54	7364		
**Peroxidases**					
Peroxidase	comp252_c0_seq1_1	95	4240	5.5E + 00	9.1E-03
	comp252_c0_seq3_1	22	994	5.5E + 00	1.3E-02
	comp252_c0_seq4_1	83	6515	6.3E + 00	4.5E-03
	comp252_c0_seq5_1	9	877	6.8E + 00	1.4E-02
	sum	208	12625		
Peroxidase 49	comp2084_c0_seq1_1	145	2649	4.3E + 00	3.0E-02
Gluthatione peroxidase	comp6217_c0_seq4_1	0	75	3.0E + 01	4.5E-02
**Dioxygenases**					
Flavanone 3-hydroxylase	comp225_c0_seq1_1	1507	20326	3.8E + 00	1.0E-02
Flavanone 3-hydroxylase	comp453_c0_seq1_1	706	16165	4.5E + 00	1.3E-02

Potential candidate cytochromes, peroxidases and dioxygenases with a significantly higher expression level in glandular trichomes. The normalized counts of 6 samples with filamentous trichomes are summed and compared with 6 glandular trichome samples. Log2-fold changes are given as well as adjusted p-values.

Comp69 was detected 6 log_2_-fold more in glandular compared to filamentous trichomes. The best BLAST-hit was with CYP81B1 [GenBank: CAA04117] from *Helianthus tuberosus*. This enzyme is functionally characterized to hydroxylate medium-chain saturated fatty acids [53]. In addition to this, CYP81B1 can be assumed to epoxidize these fatty acids [53,54]. In glandular trichomes, comp548_c0_seq1 and comp548_c0_seq2 were detected 5 log_2_-fold more than in filamentous trichomes, these contigs were annotated as P450 mono-oxygenase and showed homology with predicted sequences from the CYP82 family and CYP82C9v3 [GenBank: XP_002327091] from *Populus trichocarpa*. This CYP82 family is relatively uncharacterized [55]. A characterized enzyme from this family is CYP82G1 [GenBank: Q9LSF8] from *Arabidopsis thaliana* which is involved in homoterpene biosynthesis in which epoxidation might occur [56,57]. But this characterized protein was not present in the list of best-BLAST hits with comp548. Another cytochrome P450 significantly more expressed in glandular trichomes was comp2774. The full length of this transcript was determined by Misra *et al*. and called CIM_CYP03 (CYP72A) [GenBank: GU318227] [58]. The activity of this enzyme was tested *in vitro* with artemisinic acid, dihydroartemisinic acid, arteannuin B and artemisinin as substrates but no activity was detected [58]. Other contigs significantly more expressed in glandular trichomes were comp15043_c0_seq2 annotated as CYP76B1 and comp3673 as cytochrome P450. Comp586_c0_seq3 and comp586_c0_seq4 were annotated as a cytochrome c-type. Some other cytochromes were significantly more expressed in filamentous trichomes as shown in additional data [see Additional file 8].

Comp252 (seq1, seq3, seq4 and seq5), 2084 and 6217_c0_seq4_1 are peroxidases detected respectively 6 log_2_-fold, 4 log_2_-fold and 30 log_2_-fold more in glandular trichomes. Comp2084 was more specifically annotated as peroxidase 49 precursor and comp6217 was annotated as gluthatione peroxidase. In filamentous trichomes, two contigs annotated as peroxidases were significantly more expressed than in glandular trichomes. These contigs are comp3274 annotated as peroxidase 1 from *A. annua* and comp16324 as alkaline leaf peroxidase from *Cyanara cardunculus*.

Two dioxygenases were significantly more expressed in glandular trichomes: comp225_c0_seq1 and comp453 were both annotated as naringenin 2-oxoglutarate 3-dioxygenase (flavanone 3-hydroxylase).

### Other terpene synthases in glandular and filamentous trichomes

Several contigs corresponding to enzymes involved in the biosynthesis of other sesquiterpenoids were significantly upregulated in glandular trichomes as well. These contigs are listed in the supplementary data [see Additional file 8]. Despite a higher *germacrene-A synthase* expression in glandular trichomes, germacrene-A was only detected in biotypes without glandular trichomes [59]. This can be the effect of up-regulation of germacrene-A oxidase which further oxidizes germacrene-A [60].

In filamentous trichomes, contig comp2645 corresponding to 8-epi-cedrol synthase, was significantly upregulated [see Additional file 8]. Low expression of *8-epi-cedrol synthase* in glandular trichomes has been observed by qRT-PCR [23]. *In vitro*, recombinant 8-epi-cedrol synthase converts farnesyl diphosphate to 8-epicedrol, cedrol and minor amounts of α- cedrene and (E)-β-farnesene [61,62]. Since a higher amount of *8-epi-cedrol synthase* was detected in filamentous trichomes, these trichomes might synthesize the majority of these metabolites. Differences in (E)-β-farnesene concentration in glanded and glandless biotypes were estimated from Tellez *et al*. by correlating relative peak area to the oil content in fresh plant material [59]. Based on these estimates, the level of (E)-β-farnesene is approximately 1.8 times higher in glandless biotypes. This might correlate with the upregulated expression of 8-epi-cedrol synthase in filamentous trichomes. From α-cedrene, only trace amounts were measured in glanded and glandless biotypes [59] and the major product cedrol epimers were even not detected in extracts of *A. annua*[61,62].

Regarding diterpenoid biosynthesis, only contigs annotated as *momilactone A synthase* were significantly more expressed in glandular trichomes [see Additional file 8]. Concerning monoterpenoid biosynthesis, many monoterpenoid synthases were significantly upregulated in glandular trichomes [see Additional file 8]. This is corroborated when comparing isoprenoid contents in glanded and glandless *A. annua*[59]. In oil from glanded biotypes, monoterpenes were predominant whereas in oil from glandless biotypes monoterpenes were almost absent.

β-amyrin synthase, an enzyme that converts 2,3-oxidosqualene to the triterpene saponin β-amyrin, has been characterized in *A. annua*[63]. This enzyme was represented in the *de novo* assembly by comp33386, comp59983, comp96251 and comp23239 and these contigs were not significantly differentially expressed. Contig comp7642_c0_seq2_1 shows homology with both dammarenediol synthase and (β-)amyrin synthase and is a yet uncharacterized oxidosqualene cyclase (U_OSC). This contig was detected significantly more in filamentous trichomes [see Additional file 8].

### Lipid biosynthesis in glandular and filamentous trichomes

**Figure 4:**
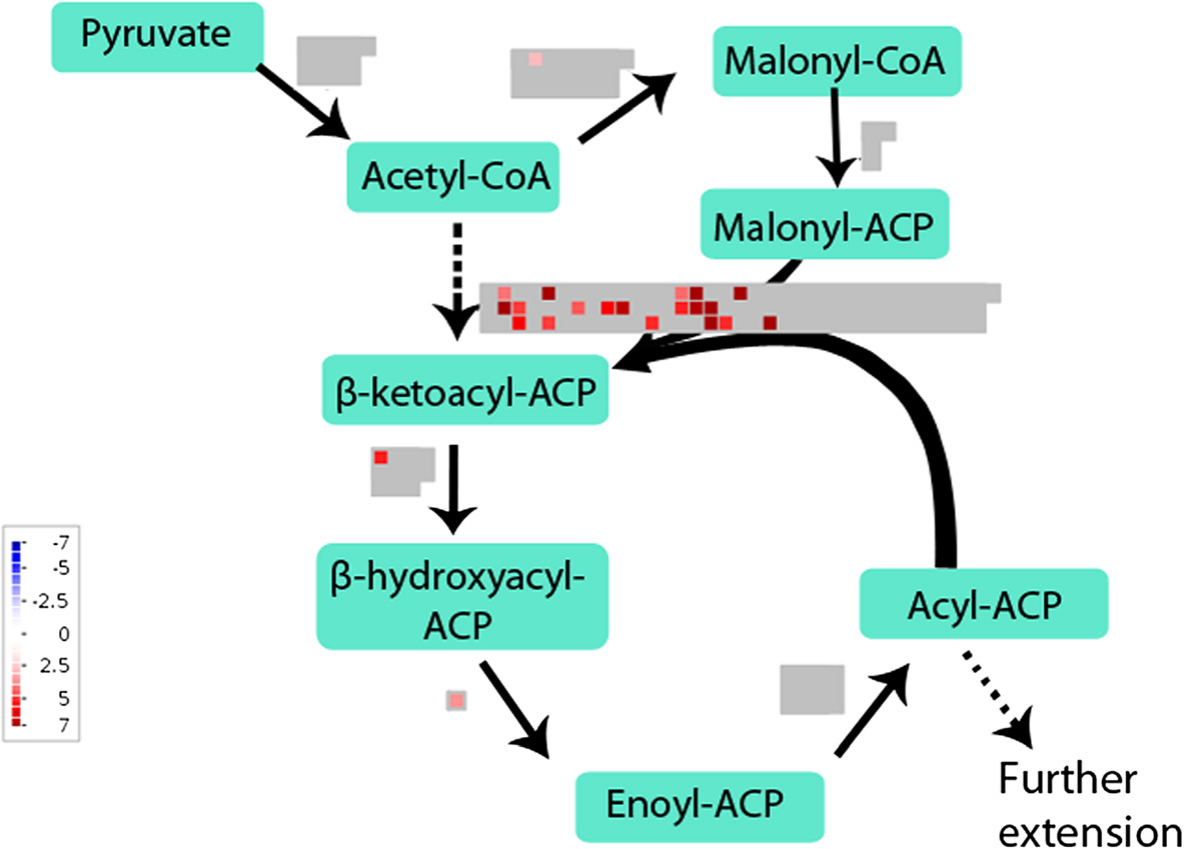
**Differentially expressed lipid biosynthesis genes in glandular and filamentous trichomes.** MapMan file with comparison of lipid biosynthesis pathways in glandular and filamentous trichomes. Significantly more expressed contigs in glandular trichomes are shown in red with colour scale to indicate the log_2_-fold change. There were no contigs with higher expression in filamentous trichomes. Grey represents contigs that were not significantly differentially expressed.

In glandular trichomes, 15% of the significantly upregulated contigs were annotated to lipid biosynthesis. Transcripts and their corresponding significantly differentially expressed contigs are listed in supplementary data [see Additional file 8]. Acetyl-CoA carboxylase converts acetyl-CoA to malonyl-CoA and was significantly upregulated in glandular trichomes as shown in Figure 4. Subsequently, malonyl CoA-acyl carrier protein transacylase converts malonyl-CoA to malonyl-ACP [64]. This transcript was not significantly upregulated. Fatty acid biosynthesis is initiated by the condensation of malonyl-ACP with acetyl-CoA by β-ketoacyl-ACP synthase III (KAS). β-ketoacyl-ACP is reduced by β-ketoacyl-ACP reductase, dehydrated by β-hydroxyacyl-ACP dehydratase and reduced by enoyl-ACP reductase to yield butyryl-ACP. The latter transcripts except enoyl-ACP reductase were significantly more expressed in glandular trichomes as shown in Figure 4. The acyl-ACP end product has two carbons more than the original acetyl molecule [64]. Similar elongation cycles are continued with condensation of malonyl-ACP and acyl-ACP and the removal of the β-ketogroup. Three types of KAS were present with different acyl chain length specificities: KASIII (C_2_ to C_4_), KASI (C_4_ to C_16_) and KASII (C_16_ to C_18_) [64]. All 3 types of *KAS* were upregulated in glandular trichomes.

Further extension of C16 and C18 to longer fatty acids requires their liberation from ACP by acyl-ACP thioesterase. Subsequently, fatty acids are exported out of the plastid to the endoplasmic reticulum [64]. The extension of fatty acids from long (C16,C18) to very long chains is catalyzed by β-ketoacyl-CoA synthase, β-ketoacyl-CoA reductase, β-hydroxyacyl-CoA dehydratase and enoyl-CoA reductase [65]. The rate-limiting step and specificity is determined by the β-ketoacyl-CoA synthase which was significantly more expressed in glandular trichomes [see Additional file 8].

*Fatty acyl-CoA reductase 1* (TFAR1) [22] was significantly more expressed in glandular trichomes. The encoded enzyme catalyzes the formation from acyl-CoA to fatty alcohols and is potentially involved in wax formation [22]. For the formation of unsaturated fatty acids, *omega-3 fatty acid desaturase* is significantly upregulated in glandular trichomes. Contigs annotated as *cyclopropane-fatty-acyl phospholipid synthase* were highly expressed in glandular trichomes. This enzyme is forming a cyclopropane ring in unsaturated fatty acyl chains [66,67]. Some contigs coding for lipid transfer proteins were significantly upregulated in glandular trichomes whereas other contigs coding for lipid transfer proteins were upregulated in filamentous trichomes [see Additional file 7].

The observed upregulation of lipid biosynthesis in glandular trichomes is in agreement with the results obtained by Tellez *et al*. [59], who measured that in glanded leaves 0.24% of fresh weight is oil and in glandless leaves only 0.06%.

### qRT-PCR

Enrichment for artemisinin-synthesis and triterpene-synthesis related transcripts in glandular and filamentous trichomes, respectively, was verified by qRT-PCR [see Additional file 9]. In mock-treated filamentous and glandular trichomes, similar expression levels were detected for *Actin2*, *PP2AA3* and *PPR protein* in both RNASeq and qRT-PCR. Similarly, with both techniques, no clear difference in *FDS* was observed between both trichome types. With qRT-PCR, the mean expression levels of *CYP71AV1, DBR2, Aldh1*, *Fatty acyl-CoA reductase 1* (*TFAR1*) and 10 candidate genes for artemisinin biosynthesis were higher in glandular trichomes whereas the transcripts corresponding to uncharacterized oxidosqualene cyclase (U_OSC) and 8-epi-cedrol synthase were more abundant in filamentous trichomes. Hence, this qRT-PCR experiment confirmed the RNASeq data.

### Apical versus sub-apical cells

**Figure 5:**
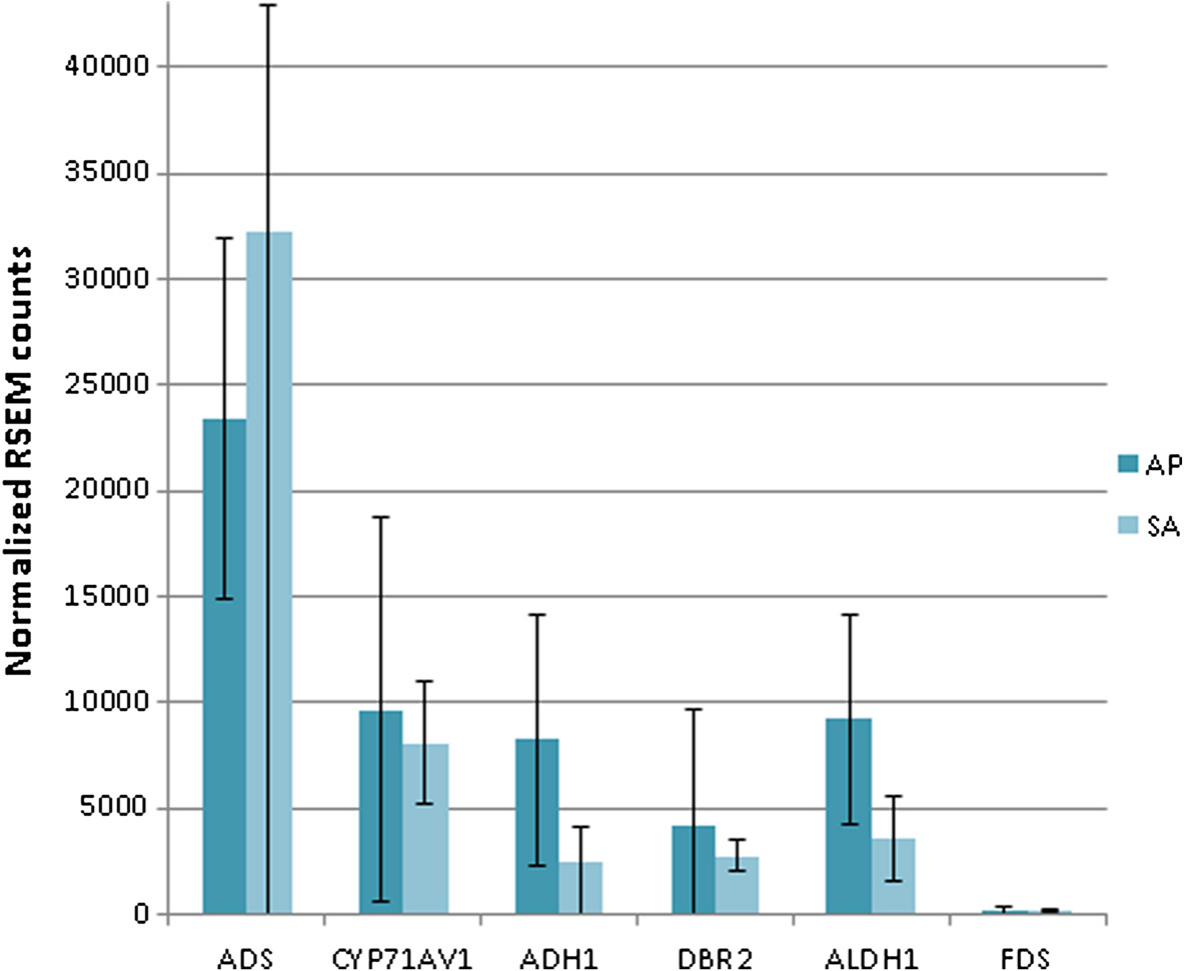
**Expression of artemisinin biosynthesis genes in apical and sub-apical cells from glandular trichomes.** Normalized counts for artemisinin biosynthesis genes in apical (AP) and sub-apical (SA) cells. Error bars represent standard deviations.

The small number of microdissected cells yielded a suboptimal amount of RNA that could be used as input for the Nugen SPIA amplification. For this reason, the amplification was biased towards the high abundant transcripts and the counts of the low abundant transcripts showed a high variation. A comparison of apical and sub-apical cells revealed no significantly differentially expressed contigs (adjusted p-value < 0.05). Contigs coding for artemisinin biosynthesis genes were detected in both apical and sub-apical cells as shown in Figure 5, which is in agreement with the findings of Olofsson *et al*. [23].

## Conclusions

On the transcript level, MEP and MVA pathways were significantly upregulated in glandular trichomes in comparison with filamentous trichomes. In addition to this, transcripts coding for the artemisinin biosynthesis pathway, other sesquiterpene biosynthesis and monoterpene pathways were predominantly expressed in glandular trichomes. Novel cytochrome-, peroxidase- and dioxygenases-encoding genes highly expressed in glandular trichomes were detected and these might be potential candidate genes for the formation of the endoperoxide bridge in artemisinin. Lipid biosynthesis pathways were highly expressed in glandular trichomes and less in filamentous trichomes. In filamentous trichomes, some specific genes from sesquiterpenoid and triterpenoid pathways such as 8-epi-cedrol synthase and oxidosqualene cyclase were detected significantly more than in glandular trichomes. Between the transcriptome of apical and sub-apical cells from glandular trichomes, no differences could be observed in the expression of artemisinin biosynthetic enzymes.

This transcriptome analysis underscores the vast metabolic capacities of *A. annua* glandular trichomes and simultaneously points to the existence of specific terpene metabolic pathways in the filamentous trichomes. Therefore, it would be interesting to examine metabolic activities in filamentous trichomes of other plant species. Besides this, it would also be interesting to characterize the potential candidate genes for artemisinin biosynthesis. If they are involved in the production of artemisinin, they can be used to produce artemisinin in yeast cells.

## Abbreviations

AACT: Acetyl-CoA C-acetyltransferase; ADS: Amorpha-4,11-diene synthase; ADH1: Alcohol dehydrogenase; ALDH1: Aldehyde dehydrogenase 1; CMK: 4-cytidine 5’-diphospho-2-C-methyl-D-erythritol kinase; CYP71AV1: Amorpha-4,11-diene monooxygenase; DBR2: Artemisinic aldehyde Δ11(13) double bond reductase; DXS: 1-deoxy-D-xylulose-5-phosphate synthase; DXR: 1-deoxy-D-xylulose-5-phosphate reductoisomerase; EST: Expressed sequence tags; FDS: Farnesyl diphosphate synthase; GO: Gene onthology; HDS: Hydroxy-2-methyl-2-(E)-butenyl 4-diphosphate synthase; HDR: Hydroxy-2-methyl-2-(E)-butenyl 4-diphosphate reductase; HMGR: 3-hydroxy-3-methyl-glutaryl coenzyme A reductase; HMGS: 3-hydroxy-3-methyl-glutaryl coenzyme A synthase; Hsp: High-scoring segment pairs; IDI: Isopentenyl diphosphate isomerase; LSU: Large subunit; JA: Jasmonic acid; KAS: β-ketoacyl-ACP synthase; MCS: 2-C-methyl-D-erythritol-2,4-cyclodiphosphate synthase; MCT: 2-C-methyl-D-erythritol-4-phosphate cytidylyltransferase; MEP: 2-C-methyl-D-erythritol 4-phosphate; MVA: Mevalonate pathway; MVK: Mevalonate kinase; PMK: Phosphomevalonate kinase; PMD: Diphosphomevalonate decarboxylase; PP2AA3: Protein phosphatase 2A subunit A3; PPR: Pentatricopeptide repeat superfamily protein; RED1: Dihydroartemisinic aldehyde reductase; RSEM: RNASeq by Expectation Maximization; SSU: Small subunit; TFAR1: Fatty acyl-CoA reductase 1; TMM: Trimmed mean of M values; U_OSC: Uncharacterized oxidosqualene cyclase.

## Competing interests

The authors declare that they have no competing interests.

## Authors’ contributions

SS planned the experimental setup, performed all the laboratory work except for the sequencing, was involved in the bioinformatics analysis, interpreted the data and wrote the manuscript. CVN performed the bioinformatics analysis and wrote part of the manuscript. MV provided technical support on the laser microscope for collecting the samples. SH helped in optimizing the library preparation and performed the cluster generation as well as the sequencing. AG participated in designing the experiment, biological interpretation of the data and writing. FVN participated in designing the experiment, the bioinformatics analysis and the writing of the manuscript. DD participated in designing the experiment and writing. All authors reviewed and approved the final manuscript.

## Supplementary Material

Additional file 1 ***Artemisia annua *** **Anamed grown in 8 h light, 16 h night.** Pictures of 7 months old *Artemisia annua* Anamed plants (A, B) grown in a growth room under 8 h light, 16 h night photoperiod. C: detail of the flower bud stage used to collect trichomes. Click here for file

Additional file 2 **Trichomes on a flower head of *A. annua.*** SEM picture of a flower head (capitulum) from *A. annua* Anamed with some bracts opened to show floret buds. On the floret buds, glandular trichomes are protruding whereas they are sunken in the capitulum bracts. Filamentous trichomes are abundantly present on the basal bracts. Click here for file

Additional file 3 **Primers for qRT-PCR.** Overview of primer sequences used for qRT-PCR. Click here for file

Additional file 4 **Transcriptome assembly of trichomes from *Artemisia annua.*** All 150,288 *de novo* assembled sequences and their contig-numbers generated with Trinity. Click here for file

Additional file 5 **Metabolite concentrations in flower heads of mock- and JA-treated plants.** Graphs showing metabolite concentrations in flower heads of *A. annua*. Artemisinin, arteannuin B, dihydroartemisinic acid and artemisinic acid levels were measured with HPLC-MS/MS in mock- and JA-treated plants. Click here for file

Additional file 6 **Metabolite concentrations in entire plant material of mock- and JA-treated plants.** Graphs showing metabolite concentrations in entire plant material of *A. annua*. Artemisinin, arteannuin B, dihydroartemisinic acid and artemisinic acid levels were measured with HPLC-MS/MS in mock- and JA-treated plants. Click here for file

Additional file 7 **Significant differences in transcriptome expression in glandular and filamentous trichomes.** List with all contigs that are significantly differentially expressed in glandular and filamentous trichomes. Click here for file

Additional file 8 **Overview from the commented differences between glandular and filamentous trichomes.** Overview of all contigs discussed in this article including calculation of log_2_-fold changes and adjusted p-values. Click here for file

Additional file 9 **qRT-PCR analysis on filamentous and glandular trichomes.** Bar chart showing qRT-PCR results on Nugen amplified material and on top the abundance of the corresponding transcripts in the RNASeq data (similar, decreased or enhanced abundance in filamentous and glandular trichomes). Of 3 biologic repeats, geometric averages from relative quantities were calculated with qbasePLUS and shown against a linear scale with as error bars the standard errors of the geometric mean. CF and CG represent mock-treated filamentous and glandular trichomes, respectively. Click here for file
