# Supplementary figures and images for: Differential transcriptome analysis of glandular and filamentous trichomes in Artemisia annua
Source: BMC Plant Biol. 2013 Dec 20;13:220. doi: 10.1186/1471-2229-13-220 (PMC3878173; doi:10.1186/1471-2229-13-220)

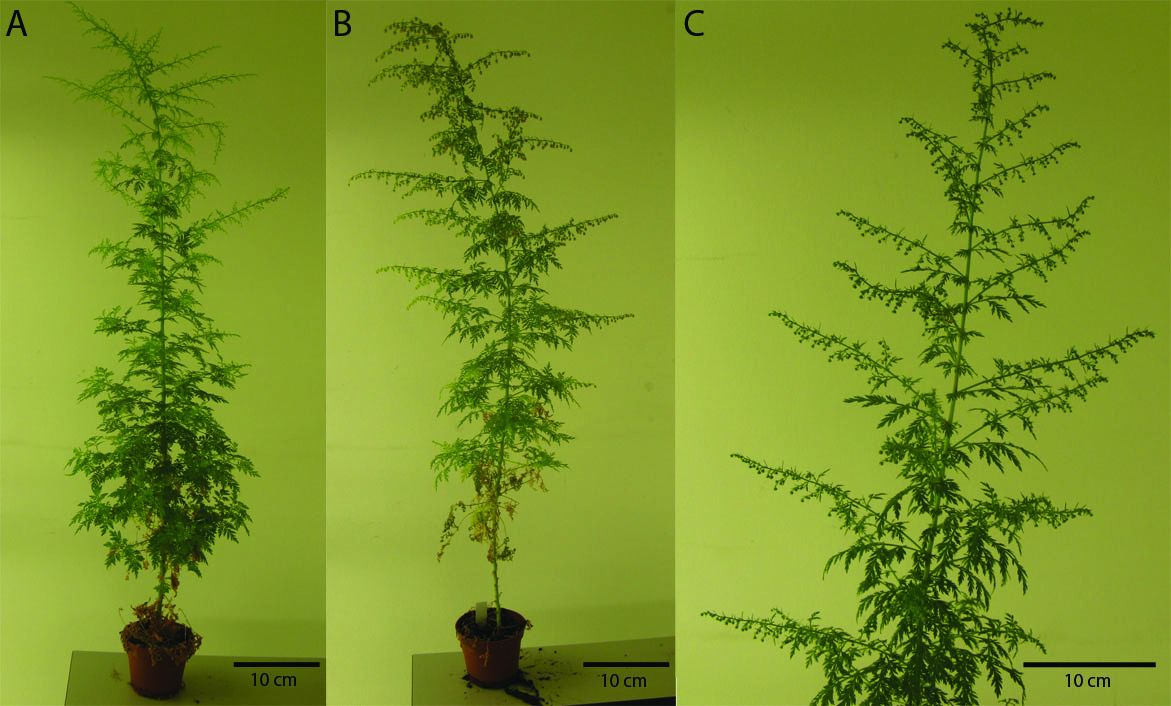

Supplement: Additional file 1 — Artemisia annua Anamed grown in 8 h light, 16 h night. Pictures of 7 months old Artemisia annua Anamed plants (A, B) grown in a growth room under 8 h light, 16 h night photoperiod. C: detail of the flower bud stage used to collect trichomes. [file 1471-2229-13-220-S1.jpeg]

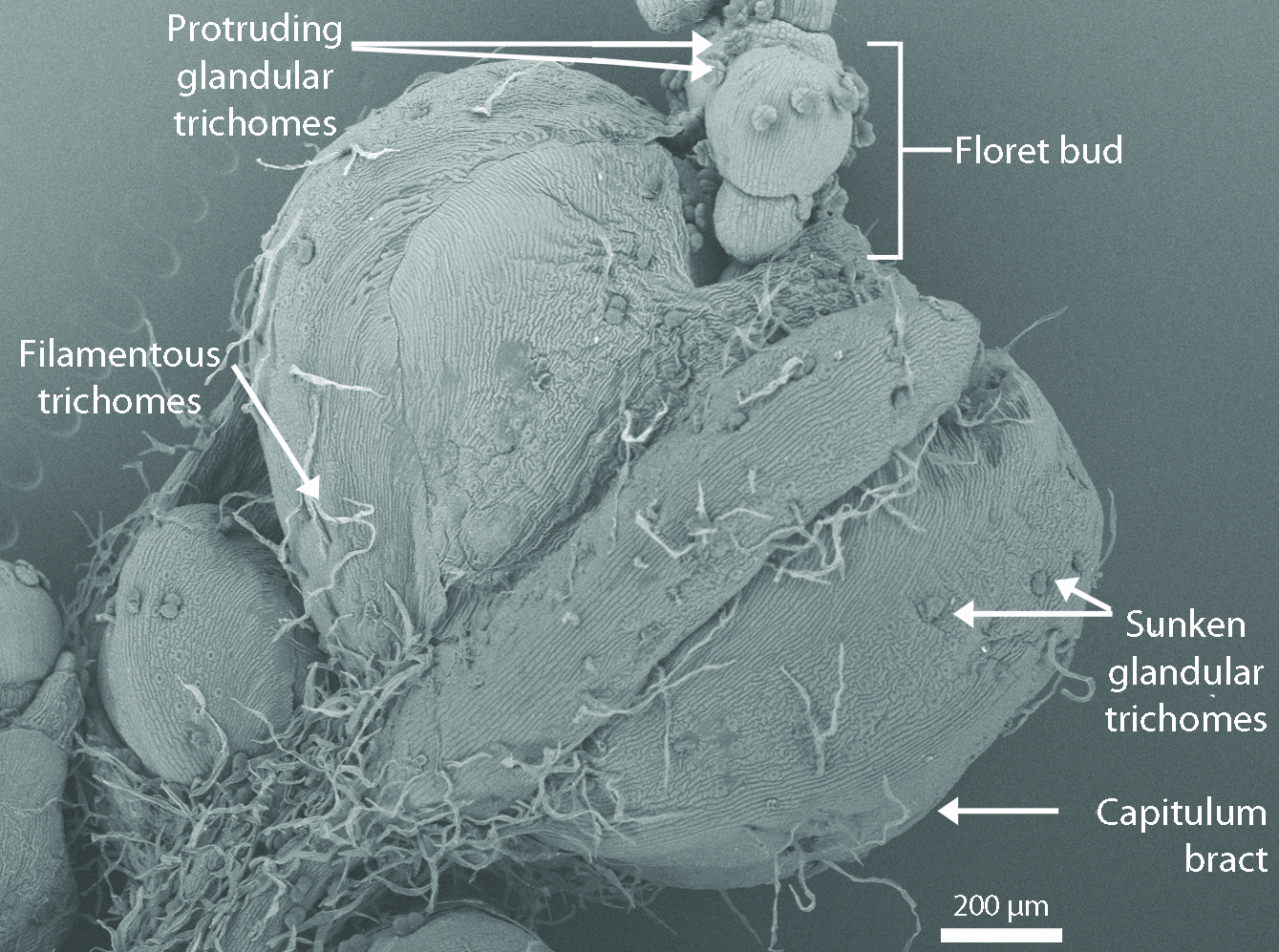

Supplement: Additional file 2 — Trichomes on a flower head of A. annua. SEM picture of a flower head (capitulum) from A. annua Anamed with some bracts opened to show floret buds. On the floret buds, glandular trichomes are protruding whereas they are sunken in the capitulum bracts. Filamentous trichomes are abundantly present on the basal bracts. [file 1471-2229-13-220-S2.tiff]

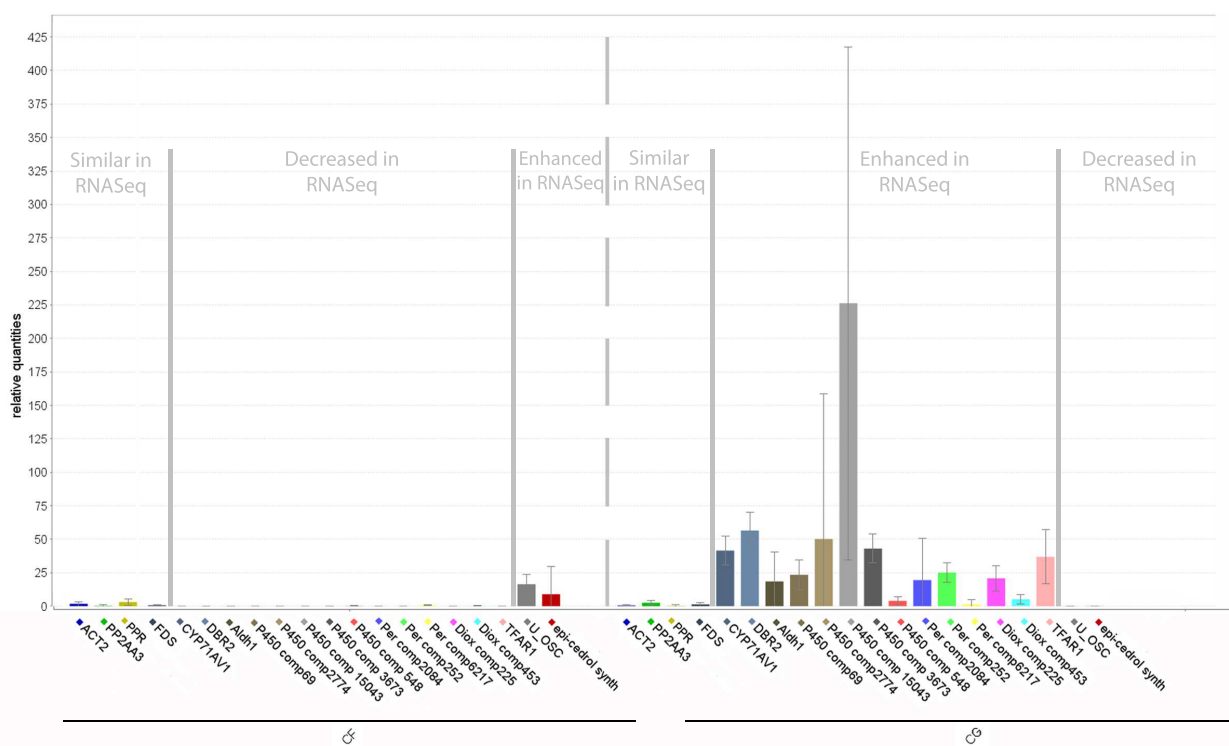

Supplement: Additional file 9 — qRT-PCR analysis on filamentous and glandular trichomes. Bar chart showing qRT-PCR results on Nugen amplified material and on top the abundance of the corresponding transcripts in the RNASeq data (similar, decreased or enhanced abundance in filamentous and glandular trichomes). Of 3 biologic repeats, geometric averages from relative quantities were calculated with qbasePLUS and shown against a linear scale with as error bars the standard errors of the geometric mean. CF and CG represent mock-treated filamentous and glandular trichomes, respectively. [file 1471-2229-13-220-S9.pdf]
