# Supplementary material for: Differential transcriptome analysis of glandular and filamentous trichomes in Artemisia annua
Source: BMC Plant Biol. 2013 Dec 20;13:220. doi: 10.1186/1471-2229-13-220 (PMC3878173; doi:10.1186/1471-2229-13-220)
Supplement: Additional file 3 — Primers for qRT-PCR. Overview of primer sequences used for qRT-PCR. [file 1471-2229-13-220-S3.pdf]

Overview of primer sequences used for qRT-PCR.

Transcripts	Primer sequences	
Actin 2	TCAGGCCGTGCTTTCTCTTT	AGATGGGCACTGTATGGGAC
PP2AA3	TACCATATACCGCACACGCC	GGGAAGTTGTTACAGCCCCA
PPR	GGGAAACTTAAGGTTGCGGT	ACCATCCCGAGAGTACCCAT
FDS	CCCGAGGTGATTGGAAAGATTG[1]	CGAATACAGCCTGAAGATTGAGAG[1]
CYP71AV1	CGAGACTTTAACTGGTGAGATTGT[2]	CGAAGCGACTGAAATGACTTTACT[2]
DBR2	ATGGAAGTGAGGAGGAAG[1]	AAACAAGGTCAGGATTCG[1]
Aldh1	TCGGAGTAGTTGGTCACATC[1]	TCACGCCATCAGGAACAC[1]
P450 comp69	CTATTGCGCTTTGGGTCACG	GTAATCGAGGGCGGTTAGCA
P450 comp2774	TTTGATTGGGGGTTGCCAGA	CATGGTTAACCCCGGCAAAA
P450 comp 15043	TTGTGGACTTGTTTGTGGCG	GTGTGCGGGTTGCGTAGAA
P450 comp 3673	AGCGAGGATCACGTTGCATT	GCGGTCACCACATGAGGATT
P450 comp 548	GCACTCCAAGAGTTGCGGAT	AGTAATTCCCGCGCTTTCAG
Per comp2084	TTCGTACAGGGCTGTGATGG	TGGGTGCGTTCTTTTCTCCT
Per comp252	CAGGTCAGACCAGCAGTTGT	CCGCGAAATCCTCATGGAAAC
Per comp6217	TTCTAGTCGACCGTGAGGGA	TTCTCGATGCTGAGTGGACTG
Diox comp225	AGGAGAAGCGGTGCAAGATT	AACTGCCCTCCACTCTTTGG
Diox comp453	GGTGGCGAAGACTTGGTCTA	CATGTCGGTATGTGGGGCTA
TFAR1	CAAACCCTTTTCAGTTCACCA[1]	ATGACAGCCTTTCCATCCTTT[1]
U_OSC	CGGTCGAGCGTCAAGAAGTA	CGCATAAGCAAATCACCGCA
epi-cedrol synthase	TTGGTTCCCATAAGGGCGAG	CGTAGGCTTGTTGCTCGCTA

References

1. Maes L, Van Nieuwerburgh FCW, Zhang YS, Reed DW, Pollier J, Casteele S, Inze D, Covello PS, Deforce DLD, Goossens A: **Dissection of the phytohormonal regulation of trichome formation and biosynthesis of the antimalarial compound artemisinin in *Artemisia annua* plants**. *New Phytologist* 2011, **189**(1):176-189.
2. Olofsson L, Lundgren A, Brodelius PE: **Trichome isolation with and without fixation using laser microdissection and pressure catapulting followed by RNA amplification: Expression of genes of terpene metabolism in apical and sub-apical trichome cells of *Artemisia annua* L**. *Plant Science* 2012, **183**:9-13.
