# Supplementary material for: Differential transcriptome analysis of glandular and filamentous trichomes in Artemisia annua
Source: BMC Plant Biol. 2013 Dec 20;13:220. doi: 10.1186/1471-2229-13-220 (PMC3878173; doi:10.1186/1471-2229-13-220)
Supplement: Additional file 5 — Metabolite concentrations in flower heads of mock- and JA-treated plants. Graphs showing metabolite concentrations in flower heads of A. annua. Artemisinin, arteannuin B, dihydroartemisinic acid and artemisinic acid levels were measured with HPLC-MS/MS in mock- and JA-treated plants. [file 1471-2229-13-220-S5.pdf]

Metabolite concentrations in flower heads of mock- and JA-treated plants.

After sampling for RNA extraction, metabolites from remaining flower heads were separately extracted by a 1 min chloroform-dip [1]. Artemisinin, arteannuin B, dihydroartemisinic acid and artemisinic acid were quantified by means of a HPLC-MS/MS method, developed by Van Nieuwerburgh *et al.*, with following modifications [1]. The mobile phase of pump A consisted of ULC-MS pure water (Biosolve, Valkenswaard, the Netherlands) with 0.1% formic acid (Biosolve) and the mobile phase of pump B was 90% ULC-MS pure acetonitrile (Biosolve) and 10% water with 0.1% formic acid. Metabolites were separated in a run of 34 minutes (40% A and 60% B for 8 minutes, linear gradient of 9 minutes to 15% A and 85% B, 100% B for 5 minutes, 40% A and 60% B for 12 minutes). The capillary voltage of the ESI-source was 2.4 kV and the flow rate of nitrogen as desolvation gas was 400 l/h. Argon was used as a collision gas at 0.9 bar. The collision energy for dihydroartemisinic acid was set at 12 eV (m/z 237→163 + 191 + 201 + 219) and for arteannuin B the collision energy was 10 eV (m/z 249→185 + 189 + 231). The internal standard used was santonin with the collision energy set at 9 eV (m/z 247→173.2 + 201.2). For the other compounds, the same settings were used as in Van Nieuwerburgh *et al.* [1]. Standards santonin (I.S.) and artemisinin were supplied by Sigma-Aldrich, arteannuin B and artemisinic acid by the Walter Reed Army Institute of Research (Washington, U.S.A.) and dihydroartemisinic acid was donated by Patrick Covello (National Research Council Canada).

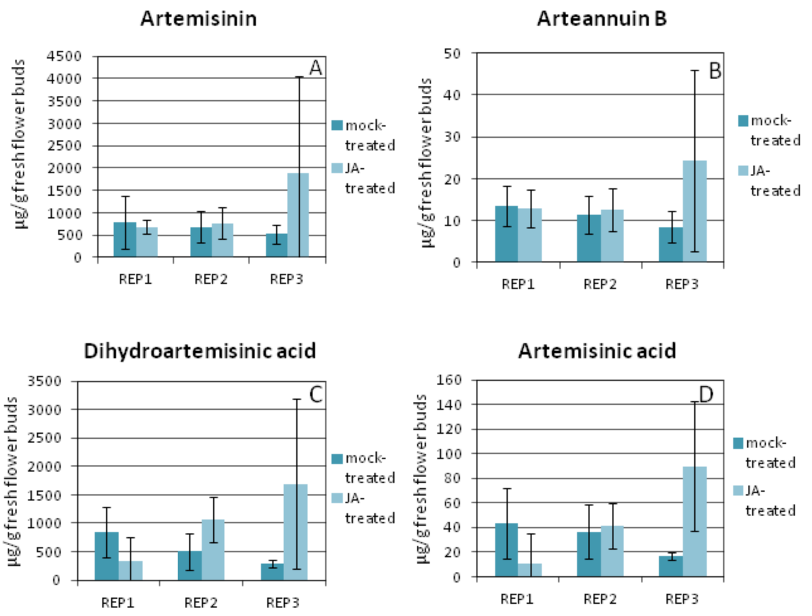

Figure 1: Metabolite concentrations in flower heads of mock- and JA-treated plants. Metabolite concentrations are shown in µg per g fresh flower head material of mock-treated and JA-treated plants. The results of all 3 repeats are shown separately. The concentration shown for each repeat is the average concentration of metabolites from 3 plants. The material for HPLC-MS/MS was derived from the same plants used for RNASeq. A: artemisinin, B: arteannuin B, C: dihydroartemisinic acid and D: artemisinic acid concentrations were measured. Equal quantities of artemisinin, arteannuin B, dihydroartemisinic acid and artemisinic acid were detected in mock- and JA-treated flower buds. Error bars represent standard deviations.

Reference

1. Van Nieuwerburgh FCW, Casteele SRV, Maes L, Goossens A, Inze D, Van Bocxlaer J, Deforce DLD: **Quantitation of artemisinin and its biosynthetic precursors in *Artemisia annua* L. by high performance liquid chromatography - electrospray quadrupole time-of-flight tandem mass spectrometry**. *Journal of Chromatography A* 2006, **1118**(2):180-187.
